# Reasons for encounter by different levels of urgency in out-of-hours emergency primary health care in Norway: a cross sectional study

**DOI:** 10.1186/s12873-017-0129-2

**Published:** 2017-06-24

**Authors:** Guttorm Raknes, Steinar Hunskaar

**Affiliations:** 1 National Centre for Emergency Primary Health Care, Uni Research Health, Kalfarveien 31, 5018 Bergen, Norway; 2 Regional Medicines and Information and Pharmacovigilance Centre (RELIS), University Hospital of North Norway, Box 79, 9038 Tromsø, Norway; 3 Department of Global Public Health and Primary Care, University of Bergen, Box 7800, 5020 Bergen, Norway

**Keywords:** After-hours care, Classification, Emergency medical services, Health services, Needs and demand, International Classification of Primary Care (ICPC), Norway, Primary health care, Reason for encounter

## Abstract

**Background:**

Frequencies of reasons for encounter (RFEs) in emergency primary care out-of-hours (OOH) services are relevant for planning of capacities as well as to target the training of staff at casualty clinics. We aimed to present frequencies of RFEs in the different organ systems, and to identify the most frequent RFEs at different urgency levels.

**Methods:**

We analyzed data on RFEs in Norwegian OOH services. International Classification of Primary Care (ICPC-2) RFE codes were recorded in all contacts to eight representative OOH casualty clinics in 2014 and 2015 covering 20 municipalities with a total population of 260 196. Frequencies of each ICPC-2 chapters and groups of ICPC-2 codes were calculated at different urgency levels.

**Results:**

Musculoskeletal, respiratory, skin, digestive and general and unspecified issues were the most frequent RFE groups. Fever was the most frequent single ICPC-2 RFE code, but was less common among the most urgent cases. Abdominal pain was the most common RFE in patients with yellow urgency level (urgent), and chest pain dominated the potentially red (potentially life threatening) cases. There was less variation in the use of ICPC-2 with increasing urgency level.

**Conclusions:**

This study identifies important differences in RFEs between urgency levels in the Norwegian OOH services. The findings provide new insight into the function of the primary health care emergency services in the Norwegian health care system, and should have implications for staffing, training and equipment in the OOH services.

**Electronic supplementary material:**

The online version of this article (doi:10.1186/s12873-017-0129-2) contains supplementary material, which is available to authorized users.

## Background

The organization of out-of-hours (OOH) emergency health care services varies considerably between countries, and this may lead to differences in what medical conditions the OOH services handle. In most countries, the population have direct access to emergency rooms in hospitals, although primary care services are available. In the public health care system in Norway, strict gatekeeping is fundamental, even for emergency health care [1]. In principle, even the most life-threatening conditions have to be assessed by primary health care doctors before admission to a hospital. Consequently, all local municipalities have to ensure access to OOH emergency primary health care (Norwegian “legevakt”) at all times for their inhabitants and all other individuals present in that area [2]. Although the OOH services should be designed to handle the most urgent cases, the services generally have a low threshold for taking in patients. Thus, most of the activity in OOH casualty clinics involves patients with non-urgent conditions. Data from a sentinel network for monitoring the activity of OOH services in Norway indicate that almost three in four contacts were classified as “green” or not urgent and only 3 percent were “red” or potentially life threatening [3]. In order to sufficiently staff and equip an OOH clinic according to demand, information about the amount and type of patients is crucial. Also for the development of standardized qualification requirements for casualty clinic staff, it is also important to know which conditions OOH nurses and doctors should expect at different urgency levels, and how often.

Demographics (age, gender) of OOH patients and the utilization of OOH-services at different times in Norway is relatively well characterized [3]. Statistics on diagnoses on reimbursement claims in the Norwegian OOH services is available [4], but its usefulness is limited by extensive use of inaccurate and general diagnose codes and the fact that the diagnosing is partly based on financial incentives. The reasons for encounter (RFE) given by patients on first contact is probably more relevant than the doctors diagnoses when planning capacities of future OOH clinics. RFEs give a better picture of the amount and types of unresolved cases that can be expected, which is important to the function of an emergency health care organization, particularly with regard to the gatekeeping performed by OOH nurses.

Knowledge about RFEs is important to characterize the role of emergency OOH services in primary health care in urgent and non-urgent situations. RFEs in OOH services have been studied previously [5, 6], but not in light of urgency level. We wanted to identify frequencies of RFEs in the different organ systems, and aimed to identify the most frequent RFEs at different urgency levels in emergency primary care.

## Methods

### Design/setting

We performed a cross sectional study based on data from the “Watchtower project”, a sentinel network of representative OOH emergency primary health care activity in Norway. The development and implementation of this project has been described in detail elsewhere [7].

Data was missing from one of the Watchtower OOH districts for the first three months of 2014 for technical reasons in connection with a reorganization. We chose to exclude the three municipalities from this district to get representative annual RFE rates, leaving Watchtower data from seven OOH-districts covering altogether 20 municipalities with a total population of 260 196, or 5.1% of Norway’s total (2014). The included OOH districts covered 15 810 km^2^, or 4.9% of Norway’s total mainland area. Average population density was 16.5 people per square kilometer. The population (2014) of the OOH-districts ranged from 4 924 to 93 121.

All patient encounters in the included Watchtower casualty clinics in 2014 and 2015 were included. Data were collected using an online data collection tool developed by the first author using Zoho Creator [8]. For every first contact trained staff (nurse or other) was instructed to record ICPC-2 (International Classification of Primary Care) code according to the official reason for encounter manual [9]. A RFE is the patient’s main complaint upon first contact. Typically, most RFEs are general symptoms or ailments, but distinct diagnoses may also be recorded if explicitly mentioned by the patient. It is important that the nurse records the patient’s own perception of the condition, even though it is unlikely or even wrong. In contrast, ICPC-2 *diagnoses* require assessment by a doctor, and are often more specific. In this study, we recorded only ICPC-2 RFEs, not diagnoses. ICPC-2 was developed by World Organization of Family Doctors (WONCA), and is used as a medical classification system universally across the Norwegian primary health care. It contains 15 chapters covering the organ systems, and two additional chapters for psychological and social problems. Personnel of all participating OOH districts received training in determining ICPC-2 RFE codes. Only codes for symptoms and complaints (codes -01 to -29), and diagnoses and diseases (codes -70 to -99), were applied. In addition, the staff recorded age and gender of patient, time and mode of contact, home municipality and type first action taken. Urgency level according to the Norwegian Index for Medical Emergency Assistance (Index) [10] was recorded.

The urgency levels are green (not urgent), yellow (urgent) and red (potentially life threatening). Red responses imply immediate ambulance dispatch and alarming of doctor on call. In yellow responses, the need for ambulance and doctor alarm is constantly assessed, whereas the green responses in many cases can be referred to a normal “in-hours” consultation [10].

### RFE groups

There is a certain overlap between several of the ICPC-2 codes, often across chapters. For example, chest pain may be coded as general chest pain (A11), heart pain (K01), pressure/tightness of heart (K02) or musculoskeletal thoracic pain (L04). The clinical relevance is therefore limited when focusing on single codes. To get a better picture of the RFE repertoire, some of the ICPC-2 RFE codes were classified into 22 groups based on consensus between the authors (Table 1).

**Table 1:** Groups of related ICPC-2 RFE codes

RFE Group	Included ICPC-2 codes
Abdominal pain	D01, D02, D06, D88
Alcohol/substance abuse/addiction	P15, P16, P17, -P18, P19
Anxiety	P01, P02, P74, P75, P79
Chest symptom/condition	A11, K01, K02, L04
Depression	P03, P76, P77, P78
Diarrhoea/Vomiting	D09, D10, D11, D70, D73
Ear symptom/condition	H01, H70, H71
Eye symptom/condition	F01, F02, F03, F15, F16, F70, F71, F72, F73, F76, F79, F85
Fears, concerns and worries	All --26 and --27. A13, A25, H15, K245, K254, W02, W21, X22, X23, X24, X25, Y24, Y25
General symptom	All -29
Head/face symptom/condition	N01, N03, N89, N90, N92, N95
Lower limbs symptom/injury/condition	L13, L14, L15, L16, L17, L73, L75, L77, L78, L89, L90, L96
Mouth/teeth symptom/condition	D19, D20, D82, D83
Neck/back symptom/condition	L01, L02,L03, L84, L85, L86
Nose/sinus symptom/condition	R07, R08, R09, R75
Respiratory infections	R74, R77, R78, R81, R82, R83
Respiratory symptom/condition	R01, R02, R03, R04, R05
Skin injury	S15, S16, S17, S18, S19
Skin itching/rash	S02, S06, S07
Throat symptom/condition	R21, R23, R72, R76
Upper limb symptom/injury/condition	L08, L09, L10, L11, L12, L72, L74, L92, L93
Urinary tract symptom/condition	U01, U02, U06, U07, U71

### Outcomes

The main outcomes are the total RFE frequencies of the different ICPC-2 chapters, both as proportion of total contacts, and as annual rates per 100 000 person-years. ICPC-2 chapter rates for each out-of-hours district were also calculated, and the range was recorded as a measure of variance. Other outcomes include frequencies of ICPC-2 chapters and RFE groups, and the identification of the 20 most frequent ICPC-2 RFE codes in the three urgency levels.

### Statistical methods

SPSS 22 and Excel 2013 were used to handle data. Counts and relative frequencies of RFEs were calculated in in addition to incidence rates for the total study population. For ICPC-2 chapters, 95% confidence intervals for proportions of relative frequencies were calculated, and the maximum and minimum incidences among the included seven OOH districts were presented. For the 20 most frequent RFE groups and codes, 95% confidence intervals for incidence (Poisson rate) with normal approximation was calculated.

### Missing data

RFE (ICPC-2 code) was not a mandatory field in the data collection tool. We therefore anticipated a relatively high number of cases with missing or unknown RFE. To assess whether there was any selection bias, average age, distribution of gender, urgency level and time of day was calculated as baseline data in cases with and without ICPC-2 RFE codes.

All other fields in the data collection tool were mandatory, but there was an “unknown” option for each piece of information, forcing the operator to actively, and not by default, indicate when information was missing. Although the operators were instructed to record all contacts, some cases unavoidably were not recorded at all. Reasons for this might be that Watchtower recordings had to be down-prioritized in extremely busy periods, or that some of them for different reasons were forgotten. It was not possible to count these omitted cases, but comparisons between extrapolations of the Watchtower data and data based on reimbursement claims, indicate that approximately 15% of cases are missing in the Watchtower database [3].

## Results

In total, 177 053 contacts were recorded from the seven included OOH districts, 86 089 in 2014 and 90 964 in 2015. ICPC-2 RFE code was recorded in 160 215 (90.5%) of cases.

16 738 (9.5%) of the records lacked information about ICPC-2 RFE, and 359 (0.2%) lacked information about urgency level. Of these, 232 (0.1%) had neither ICPC-2 RFE code nor urgency level.

In Table 2 characteristics of patients with recorded ICPC-2RFE code is compared with those with unknown RFE. The differences were small, but the proportion of green cases and the proportion recorded at night was higher among encounters without known ICPC-2 RFE code. There was a higher number of missing ICPC-2 RFE codes when information about gender, urgency level or time of day was also missing.

**Table 2:** Baseline data ± standard error of mean (SEM) in contacts with or without known ICPC-2 RFE

	ICPC-2-code known	ICPC-2-code unknown
N	157 926	16 738
Mean Age (± SEM)	36.3 (26.2 ± 0.1)	36.8 (26.5 ± 0.2)
Age unknown, N (% ± SEM)	2 361 (1.5 ± <0.1)	1 308 (8.8 ± 0.1)
Gender, N (% ± SEM)		
Female	87 119 (54.3 ± 12.5)	9 064 (54.2 ± 18.0)
Male	72 859 (45.4 ± 12.5)	7 526 (45.0 ± 16.5)
Gender unknown	337 (0.2 ± <0.1)	148 (0.9 ± 2.4)
Urgency level, N (% ± SEM)		
Green	113 221 (70.6 ± 11.3)	12 749 (76.2 ± 21.1)
Yellow	41 836 (26.1 ± 11.1)	3 296 (19.7 ± 11.0)
Red	5 131 (3.2 ± <0.1)	461 (2,8 ± 4.2)
Urgency level unknown	127 (0.1 ± <0.1)	232 (1.4 ± <0.1)
Time of day, N (% ± SEM)		
Daytime (8:00-15:59)	60 944 (38.0 ± 12.2)	6 617 (39.5 ± 15.5)
Evening (16:00-22:59)	77 702 (48.5 ± 12.6)	7 614 (45.5 ± 16.6)
Night (23:00-7:59)	21 669 (13.5 ± 8.7)	2 502 (14.9 ± 9.7)
Time of day unknown	0 (0.0 ± 0)	5 (<0.1 ± <0.1)

The distributions of ICPC-2 chapters at different urgency levels are presented in Additional file 1: Table S1, Additional file 2: Table S2, Additional file 3: Table S3 and Additional file 4: Table S4. The number of general and unspecified (chapter A) was high for all urgency levels, especially among the most acute (red) cases, where 34% of RFEs were from chapter A. In red urgency level, 78% of the cases had a RFE from one of the five most frequent ICPC-2 chapters, compared to 65% of green records and 66% of yellow.

Cases with yellow urgency level had the highest proportion of digestive issues (chapter D, 12%). The proportions of neurological (chapter N) and circulatory (chapter K) issues were two- and three-fold higher in yellow cases compared to green respectively, but proportion of chapter H (ear) was less than a fifth.

Eighteen percent of RFEs with red urgency level were circulatory (chapter K) in contrast to only 1.3% of the green and 3.9% of the yellow RFEs. Compared to green cases, patients with red urgency level had a 3 times higher proportion of neurological cases (chapter N). Chapters L (musculoskeletal), U (urological) and F (eye) had a considerably lower proportion of red cases compared with green and yellow. Psychological/psychiatric RFEs (chapter P) were less frequent in green contacts than in yellow and red, where they constituted approximately one in twenty cases.

As can be seen from Additional file 1: Table S1, Additional file 2: Table S2, Additional file 3: Table S3 and Additional file 4: Table S4, there was a considerable variation in incidence rates of the different ICPC-2 chapters from one OOH district to another. The cardiovascular incidence rate ranged from 695 to 1 555 cases per 100 000 person-years. The differences between OOH clinics were most pronounced in the red urgency level, where the variation was 3.7-fold in chapter R (respiratory), 3.8-fold in chapter C (circulatory), 4.7-fold in chapter A (general/unspecified), 5.1-fold in chapter D (digestive), and 5.9-fold in chapter N (neurological).

The 20 most frequent RFEs in the three urgency levels are presented in Tables 3, 4 and 5. These rankings include both RFE groups and ICPC-2 RFE codes that were not assigned a group. The 20 most frequent individual ICPC-2 RFE codes in the three urgency levels are presented in Table 6.

**Table 3:** The 20 most frequent reasons for encounter at green urgency level (not urgent). RFE groups (G) and non-grouped ICPC-2 RFE codes ranked together. Counts, incidences, absolute and cumulative proportions

				Incidence (per 100 000 person-years)		Proportion of green RFEs	
Rank	Reason for encounter (RFE)	ICPC-2 or RFE group (G)	N		95% CI	%	Cumulative %
1	General symptom	G	10 031	1 928	(1 874 to 1 981)	8.0	8.0
2	Lower limbs symptom/injury/condition	G	8 107	1 558	(1 510 to 1 606)	6.4	14.4
3	Respiratory symptom/condition	G	7 980	1 533	(1 486 to 1 581)	6.3	20.7
4	Fever	A03	6 841	1 315	(1 271 to 1 359)	5.4	26.1
5	Upper limb symptom/injury/condition	G	6 606	1 269	(1 226 to 1 313)	5.2	31.3
6	Abdominal pain	G	5 510	1 059	(1 019 to 1 098)	4.4	35.7
7	Urinary tract symptom/condition	G	5 158	991	(953 to 1 029)	4.1	39.8
8	Fears, concerns and worries	G	4 826	927	(890 to 964)	3.8	43.6
9	Skin injury	G	4 592	882	(846 to 919)	3.6	43.4
10	Throat symptom/condition	G	4 096	787	(753 to 821)	3.3	46.7
11	Neck/back symptom/condition	G	3 560	684	(652 to 716)	2.8	49.5
12	Eye symptom/condition	G	3 495	672	(640 to 703)	2.8	52.3
13	Ear symptom/condition	G	2 658	511	(483 to 538)	2.1	54.4
14	Skin itching/rash	G	2 572	494	(467 to 521)	2.0	56.4
15	Diarrhoea/Vomiting	G	2 447	470	(444 to 497)	1.9	58.3
16	Head/face symptom/condition	G	2 028	390	(366 to 414)	1.6	59.9
17	No disease	A97	1 659	319	(297 to 340)	1.3	61.2
18	Respiratory infections	G	1 455	280	(259 to 300)	1.2	62.4
19	Anxiety	G	1 389	267	(247 to 287)	1.1	63.5
20	Mouth/teeth symptom/condition	G	1 321	254	(234 to 273)	1.0	64.5
	Other		28 314	5 441	(5 351 to 5 531)	22.5	87.0
	Unknown		12 749	2 450	(2 390 to 2 510)	10.1	97.1
	All		125 970	24 207	(24 018 to 24 396)	100.0	100.0

**Table 4:** The 20 most frequent reasons for encounter at yellow urgency level (urgent). RFE groups and non-grouped ICPC-2 RFE codes ranked together. Counts, incidences, absolute and cumulative proportions

				Incidence (per 100 000 person-years)		Proportion of yellow RFEs	
Rank	Reason for encounter (RFE)	ICPC-2 or RFE group (G)	N		95% CI	%	Cumulative %
1	Abdominal pain	G	4 036	776	(742 to 809)	8.9	8.9
2	Respiratory symptom/condition	G	3 543	681	(649 to 713)	7.9	16.8
3	Skin injury	G	2 967	570	(541 to 599)	6.6	23.4
4	Lower limbs symptom/injury/condition	G	2 890	555	(527 to 584)	6.4	29.8
5	General symptom	G	2 877	553	(524 to 581)	6.4	36.1
6	Upper limb symptom/injury/condition	G	2 660	511	(484 to 539)	5.9	42.0
7	Fever	A03	1 692	325	(303 to 347)	3.7	45.8
8	Chest pain/symptom/condition	G	1 665	320	(298 to 342)	3.7	49.5
9	Neck/back symptom/condition	G	1 388	267	(247 to 287)	3.1	52.6
10	Head/face symptom/condition	G	1 102	212	(194 to 229)	2.4	55.0
11	Urinary tract symptom/condition	G	963	185	(169 to 202)	2.1	57.1
12	Fears, concerns and worries	G	1 005	193	(151 to 182)	2.2	59.0
13	Eye symptom/condition	G	721	139	(124 to 153)	1.6	60.6
14	Throat symptom/condition	G	665	128	(114 to 142)	1.5	62.1
15	Depression	G	561	108	(95 to 120)	1.2	63.4
16	Diarrhoea/vomiting	G	555	107	(94 to 119)	1.2	64.6
17	Vertigo/dizzyness	N17	535	103	(90 to 115)	1.2	65.8
18	Alcohol/substance abuse/addiction	G	457	88	(76 to 99)	1.0	66.8
19	Anxiety	G	441	85	(74 to 96)	1.0	67.8
20	Fainting/syncope	A06	432	83	(72 to 94)	1.0	68.7
	Other		10 819	2 079	(2 024 to 2 134)	24.0	92.7
	Unknown		3 158	633	(603 to 664)	7.3	100.0
	All		45 132	8 673	(8 560 to 8 786)	100.0	100.0

**Table 5:** The 20 most frequent reasons for encounter at red urgency level (potentially life threatening). RFE groups and non-grouped ICPC-2 RFE codes ranked together. Counts, incidences, absolute and cumulative proportions

				Incidence (per 100 000 person-years)		Proportion of red RFEs	
Rank	Reason for encounter (RFE)	ICPC-2 or RFE group (G)	N		95% CI	%	Cumulative %
1	Chest pain/symptom/condition	G	1 413	272	(252 to 292)	25.3	25.3
2	Respiratory symptom/condition	G	495	95	(83 to 107)	8.9	34.1
3	General symptom	G	261	50	(42 to 59)	4.7	38.8
4	Coma	A07	231	44	(36 to 52)	4.1	42.9
5	Abdominal pain	G	216	42	(34 to 49)	3.9	46.8
6	Fainting/syncope	A06	186	36	(28 to 43)	3.3	50.1
7	Stroke	K90	170	33	(26 to 40)	3.0	53.1
8	Trauma/injury	A80	163	31	(25 to 38)	2.9	56.1
9	Convulsion/seizure	N07	130	25	(19 to 31)	2.3	58.4
10	Alcohol/substance abuse/addiction	G	111	21	(16 to 27)	2.0	60.4
11	Allergic reaction	A92	102	20	(14 to 25)	1.8	62.2
12	Depression	G	87	17	(12 to 22)	1.6	63.8
13	Head/face symptom/condition	G	75	14	(10 to 19)	1.3	65.1
14	Fears, concerns and worries	G	71	14	(9 to 18)	1.3	66.4
15	Paralysis/weakness	N18	70	13	(9 to 18)	1.3	67.6
16	Lower limb symptom/injury/condition	G	68	13	(9 to 17)	1.2	68.8
17	Neck/back symptom/condition	G	61	12	(8 to 16)	1.1	69.9
18	Poisoning medical agent	A84	53	10	(6 to 14)	0.9	70.9
19	Fever	A03	50	10	(6 to 13)	0.9	71.8
20	Skin injury	G	43	8	(5 to 12)	0.8	72.5
	Other		1 075	207	(189 to 224)	19.2	91.8
	Unknown		461	89	(77 to 100)	8.2	100.0
	All		5 592	1 075	(1 035 to 1 114)	100.0	100.0

**Table 6:** Incidence (per 100 000 person-years) of the 20 most frequent single ICPC-2 RFE codes at different urgency levels

Rank #	Green (not urgent)			Yellow (urgent)			Red (potentially life threatening)		
	ICPC-2 code and description		Incidence	ICPC-2 code and description		Incidence	ICPC-2 code and description		Incidence
1	A03	Fever	1 305	D01	Abdominal pain, general	633	A11	Chest pain	164
2	R05	Cough	865	S18	Laceration/cut	621	K01	Heart pain	103
3	D01	Abdominal pain, general	841	R02	Dyspnoea	357	R02	Dyspnoea	55
4	R21	Throat symptom	759	A03	Fever	325	A07	Coma	44
5	S18	Laceration/cut	662	A11	Chest pain	209	A06	Fainting/syncope	36
6	L17	Foot/toe symptom	573	N01	Headache	186	D01	Abdominal pain, general	35
7	U71	Cystitis/Lower UTI	535	L02	Back symptom	160	K90	Stroke	33
8	L12	Hand/finger symptom	496	L12	Hand/finger symptom	149	A80	Trauma/injury	31
9	H01	Ear pain	379	L09	Arm symptom	132	R04	Breathing problem, other	30
10	L02	Back symptom	373	L16	Ankle symptom	129	N07	Convulsion/seizure	25
11	A29	General symptom	367	L17	Foot/toe symptom	127	A92	Allergic reaction	20
12	A13	Fear medical treatment	336	R21	Throat symptom	124	P77	Suicide/suicide attempt	15
13	N01	Headache	334	R05	Cough	110	N01	Headache	14
14	A97	No disease	319	D06	Abdominal pain, localized	105	N18	Paralysis/weakness	13
15	L16	Ankle symptom	319	N17	Vertigo/dizziness	103	K84	Heart disease, other	13
16	A27	Fear of other diseases	305	R29	Respiratory symptom	101	P16	Acute alcohol abuse	12
17	R02	dyspnea	288	L14	Leg/thigh symptom	90	A29	General symptom	12
18	F29	Eye symptom/complaint, other	284	A29	General symptom	85	K29	Cardiovascular symptom	11
19	L15	Knee symptom	280	R04	Breathing problem	84	A84	Poisoning medical agent	10
20	L09	Arm symptom	274	A06	Fainting/syncope	83	A03	Fever	10
Unknown ICPC-2			2 450			633			89
Total			24 207			8 673			1 075

General symptoms, limb issues, respiratory issues and fever were the most frequent non-urgent green RFEs (Table 3). In yellow cases, abdominal pain was the number one RFE (Table 4). Skin injuries constituted 6.6% of yellow encounters compared to 3.6% of green and 0.8 of red. Among the potentially life-threatening cases, chest pain dominated with 25% of RFEs. There were several RFEs that had significant relative high incidences only in the red urgency level (Table 5). Stroke (K90) is one example. When combined with ICPC-2 RFE codes N18 (paralysis/weakness) and N19 (speech disorder), such a stroke-related RFE group would be the fourth most frequent RFE with 249 encounters per 100 000 person-years.

The most frequent single ICPC-2 RFE codes for each urgency level are presented in Table 6. There was less variation in the use of codes with increasing emergency level. The 20 most frequent ICPC-2 RFE codes accounted for 41% of the green cases compared to 45% of the yellow and 64% of the red. Fever (A03) was the most common green RFE, followed by cough (R05) and abdominal pain (D01). In the yellow urgency level, abdominal pain (D01) and laceration/cut (S18) dominated. Dyspnea (R02) was the third and fever (A03) the fourth most common yellow code. Among the red cases, pain in the chest was the most frequent RFE; A11 (unspecified chest pain) and K01 (heart pain) were by far the two most common red codes, followed by shortness of breath/dyspnea (R02). There were also more neurological ICPC-2 RFE codes (including K90 - stroke/cerebrovascular accident) on the red urgency level top-20 list. Notably, abdominal pain (D01) was the 7^th^ and fever (A03) the 20^th^ most frequent red ICPC-2 RFE code.

## Discussion

Musculoskeletal and general and unspecified issues were the most frequent RFEs in this study. Chapter L and chapter A combined constituted 32.4% of all RFEs, or 110 OOH patients per 1 000 person-years. Respiratory, skin and digestive were also common. General symptoms, respiratory and lower limb issues in addition to fever were the most frequent RFE among the non-urgent (green) cases, and abdominal pain (D01) in the urgent (yellow) ones. Among potentially life threatening cases chest pain dominated. In red urgency level, 25.3% of patients had chest pain related ICPC-2 RFE codes. There was less variation in the use of ICPC-2 with increasing urgency level.

This is the first published study on RFE in Norwegian OOH emergency primary health care. Our institution publishes yearly reports based on all reimbursement claims from doctors in Norwegian OOH services. These claims contain ICPC-2 diagnoses [4]. The three most frequent chapters in reimbursement claims in 2014 were Chapter A (general and unspecified, 24.1%), Chapter R (respiratory, 15.2%) and Chapter L (musculoskeletal, 13.9%), in line with the results of this study. The most frequent ICPC-2 codes in the reimbursement statistics were A99 (general disease, not otherwise specified), R74 (upper respiratory infection, acute), U71 (cystitis/urinary infection) and D01 (abdominal pain). The ICPC-2 frequencies in these reimbursement-based statistics are not directly comparable to the results of this study; mainly because the codes are diagnoses codes set by a doctor, not RFEs set by nurse. The coding might also be biased or less precise (more unspecified codes) because the ICPC-2 code is a prerequisite for the reimbursement, which means that the coding is partly motivated by financial incentives. There is also no information about urgency level in the reimbursement claims.

In a recent study on telephone contacts to Danish OOH primary care [5], fever was the most frequent RFE. Direct comparison with our study is challenging, since no incidences were presented. Fever constituted a higher proportion of all contacts (10%) than in our results (5.4% of green contacts). In addition, general stomach pain, cough, ear pain and throat symptoms were among the top five Danish RFEs, these were also among the most frequent in our material.

In a study from Switzerland comparing walk-in patients seeking services at an emergency department with a OOH general practitioner cooperative, the frequencies of musculoskeletal (L), general and unspecified (A) and urology (U) were similar to our findings [6]. Respiratory (R), digestive (D) and psychology (P) chapters were considerably more frequent and skin (S) less frequent in the Swiss GP OOH service compared to our findings. Some of the differences could be due to better direct availability of other alternative emergency health care services in the less gatekeeping Swiss system.

ICPC-2 RFE codes have been analyzed in daytime outpatients in general internal medicine department of a small community hospital in Japan [11]. Here, there was less variation in ICPC-2 RFE codes. The 20 most common RFEs accounted for 74% of all cases in the Japanese hospital, considerably more than for all urgency levels in our material. Similar to our findings, R05 (cough) and A03 (fever) were the most common RFEs.

In a study of the epidemiology of medical emergency contacts outside hospitals in Norway [12], ICPC-2 RFEs were retrospectively assigned to cases classified as potentially life threatening. Similar to red urgency level in this study, cardiovascular events were most frequent, with an incidence of 680 per 100 000 person-years. The 2.5-fold higher incidence compared to our results, can be attributed to the fact that this study was based on data from emergency medical dispatch centers (EMCC), which are not a part of the primary care. Both respiratory and digestive issues were 2.4 times more frequent in this EMCC study compared to our OOH-data.

The main strength of this study is the high number of observations. Also, there were no competing OOH services to the casualty clinics in the investigated areas, which means that we in principle we captured all OOH activity. All relevant cases should thus have been recorded, and this means that the observed incidence rates are trustworthy. RFEs have not previously been systematically recorded in Norwegian OOH services. From previous reports, we know that the quality of the Watchtower data is good. In 2014 only 1.7% of records were missing at least one piece of information. Unavoidably, some of the contacts at the Watchtower clinics are not captured, mostly during periods of high workload. It is not possible to estimate the extent of missing records in our system. This is a potential source of bias, but we believe this is a minor problem since missing cases in the Watchtower project in general are relatively infrequent (estimated 15%), and since our findings are in line with RFE frequencies in other studies. High workload and delay due to technical problems in the web-based ICPC-2 module probably also contributed to isolated missing ICPC-2 RFE codes. Despite missing information, we believe that the ICPC-2 frequencies are representative and with insignificant bias. Less than one in ten records were without information on RFE, and the differences between records with and without ICPC-2 RFE codes were negligible.

We observed large deviations in RFE incidences between individual OOH clinics. Divergent ICPC-2 RFE coding practices and local differences in the organization of emergency health services are possible explanations. The variation may also reflect significant differences in OOH utilization, which we previously have shown are associated with differences in travel distance [13].

We find it important to stress that we have recorded RFEs, not diagnoses. The recorded ICPC-2 RFE codes are based exclusively on what the patients present upon first contact, they are not the result of an examination by a doctor. The RFEs do therefore not necessarily reflect the actual morbidity of the population. RFEs are often less precise than diagnoses. Symptoms, signs and general contact reasons dominate, and such RFEs provide a good basis for triage and limit bias in the following diagnostic process. The Reason-for-Encounter mode of the ICPC has proven reliable, adequate, and feasible [14].

Originally, the Watchtowers constituted a representative sample of the Norwegian OOH services [7]. It could be questioned whether results from the projects still are generalizable in 2014 and 2015, but we believe it gives a good picture of the situation in Norway. The Norwegian OOH services (“legevakt”) system has important characteristics that differ from OOH services in most other countries [2]. The compulsory gatekeeping function is one factor that may contribute to significant differences, especially among the most urgent cases.

### Implications

The results add important knowledge about what frequencies of different issues the staff at Norwegian OOH clinics can expect. The presented incidences may be useful in the planning of how future OOH clinics should be staffed and equipped, and give important clues to which topics that should be given priority in training of nurses and other staff. OOH services are especially suited to handle cases with yellow emergency level, and the high frequency of abdominal pain in this group indicate that this should be emphasized in the education of OOH staff. There is potential for more research on RFEs in Norwegian OOH services, for example in different subgroups of patients. In addition, it would be interesting to compare the incidences in this study with other parts of the health care system, like hospitals and ambulances.

## Conclusions

Musculoskeletal, respiratory, skin, digestive and general and unspecified issues were the most frequent RFEs in this study on Norwegian OOH services. Fever was the most frequent single RFE. Abdominal pain was the most common RFE with yellow urgency level, and chest pain dominated the potentially life threatening cases.

This study identifies important differences in RFEs between urgency levels in the Norwegian OOH services. The findings provide new insight into the function of the primary health care emergency services in the Norwegian health care system, and should have implications for training, equipment and staffing of the OOH services.

## Additional files

Additional file 1: Table S1. Reasons for encounter, ICPC-2 chapters, all urgency levels. Counts, proportions. Total, minimum and maximum incidence in individual OOH casualty clinics. (PDF 79 kb)

Additional file 2: Table S2. Reasons for encounter, ICPC-2 chapters, green urgency level (not urgent). Counts, proportions. Total, minimum and maximum incidence in individual OOH casualty clinics. (PDF 80 kb)

Additional file 3: Table S3. Reasons for encounter, ICPC-2 chapters, yellow urgency level (urgent). Counts, proportions. Total, minimum and maximum incidence in individual OOH casualty clinics. (PDF 80 kb)

Additional file 4: Table S4. Reasons for encounter, ICPC-2 chapters, red urgency level (life threatening). Counts, proportions. Total, minimum and maximum incidence in individual OOH casualty clinics. (PDF 79 kb)

## Abbreviations

GP: General practitioner
ICPC-2: International classification of primary care
OOH: Out of hours
RFE: Reason for encounter
