# Supplementary material for: Reasons for encounter by different levels of urgency in out-of-hours emergency primary health care in Norway: a cross sectional study
Source: BMC Emerg Med. 2017 Jun 24;17:19. doi: 10.1186/s12873-017-0129-2 (PMC5483255; doi:10.1186/s12873-017-0129-2)
Supplement: Supplementary file 1 — Reasons for encounter, ICPC-2 chapters, all urgency levels. Counts, proportions. Total, minimum and maximum incidence in individual OOH casualty clinics. (PDF 79 kb) [file 12873_2017_129_MOESM1_ESM.pdf]

Additional table 1: Reasons for encounter, ICPC-2 chapters. All urgency levels. Counts, proportions. Total, minimum and maximum incidence in individual OOH casualty clinics.

		Proportion of all RFEs		Incidence (per 100 000 person-years)		
**ICPC-2 Chapter (RFE)**	**N**	**%**	(95% CI)	**All OOH clinics**	**Min**	**Max**
**A – General and unspecified**	28 741	16.2	(16.1 to 16.4)	5 523	4 489	12 216
**L – Musculoskeletal**	28 710	16.2	(16.0 to 16.4)	5 517	4 610	7 654
**R – Respiratory**	21 775	12.3	(12.1 to 12.5)	4 184	3 188	7 813
**D – Digestive**	17 732	10.0	(9.9 to 10.2)	3 407	2 839	5 365
**S – Skin**	17 365	9.8	(9.7 to 9.9)	3 336	2 834	7 147
**U – Urology**	9 470	5.3	(5.2 to 5.5)	1 819	1 326	3 716
**N – Neurological**	7 468	4.2	(4.1 to 4.3)	1 435	1 266	2 244
**P – Psychological**	7 232	4.1	(4.0 to 4.2)	1 389	1 171	2 031
**F – Eye**	6 886	3.9	(3.8 to 4.0)	1 323	1 004	2 307
**K – Circulatory**	4 461	2.5	(2.4 to 2.6)	857	695	1 555
**H – Ear**	4 294	2.4	(2.4 to 2.5)	825	664	1 635
**W – Pregnancy, childbirth, family planning**	1 719	1.0	(0.9 to 1.0)	330	162	711
**X – Female genital system and breast**	1 360	0.8	(0.7 to 0.8)	261	145	518
**Y – Male genital system**	971	0.5	(0.5 to 0.6)	187	113	254
**Z – Social problems**	833	0.5	(0.4 to 0.5)	160	51	271
**T – Endocrine, metabolic and nutritional**	787	0.5	(0.4 to 0.5)	169	114	396
**B – Blood, lymphatics, spleen**	420	0.2	(0.2 to 0.3)	81	54	163
**Unknown**	16 738	9.5	(9.3 to 9.6)	3 216	856	9199
**All encounters**	177 053	100.0		34 023	28 945	52 732
