# Supplementary material for: Reasons for encounter by different levels of urgency in out-of-hours emergency primary health care in Norway: a cross sectional study
Source: BMC Emerg Med. 2017 Jun 24;17:19. doi: 10.1186/s12873-017-0129-2 (PMC5483255; doi:10.1186/s12873-017-0129-2)
Supplement: Supplementary file 2 — Reasons for encounter, ICPC-2 chapters, green urgency level (not urgent). Counts, proportions. Total, minimum and maximum incidence in individual OOH casualty clinics. (PDF 80 kb) [file 12873_2017_129_MOESM2_ESM.pdf]

Additional table 2: Reasons for encounter, ICPC-2 chapters. Green urgency level (not urgent). Counts, proportions. Total, minimum and maximum incidence in individual OOH casualty clinics.

	Proportion of green RFEs			Incidence (per 100 000 person-years)		
**ICPC-2 Chapter (RFE)**	**N**	**%**	(95% CI)	**All OOH clinics**	Min	Max
**L – Musculoskeletal**	20 619	16.4	(16.2 to 16.6)	3 962	3 445	5 751
**A – General and unspecified**	20 372	16.2	(16.0 to 16.4)	3 915	3 127	8 865
**R – Respiratory**	15 814	12.6	(12.4 to 12.7)	3 039	2 366	6 274
**S – Skin**	12 822	10.2	(10 to 10.3)	2 464	2 014	5 006
**D – Digestive**	11 878	9.4	(9.3 to 9.6)	2 283	1 901	3 615
**U – Urology**	7 317	5.8	(5.7 to 5.9)	1 406	1 057	3 262
**F – Eye**	5 518	4.4	(4.3 to 4.5)	1 060	774	2 045
**P – Psychological**	4 681	3.7	(3.6 to 3.8)	900	728	1 391
**N – Neurological**	4 102	3.3	(3.2 to 3.4)	788	657	1 240
**H – Ear**	4 001	3.2	(3.1 to 3.3)	769	619	1 594
**K – Circulatory**	1 668	1.3	(1.3 to 1.4)	321	227	803
**W – Pregnancy, childbirth, family**	1 272	1.0	(1.0 to 1.1)	244	132	437
**X – Female genital system and breast**	1 057	0.8	(0.8 to 0.9)	203	117	426
**Y – Male genital system**	686	0.5	(0.5 to 0.6)	132	75	198
**T – Endocrine, metabolic and nutritional**	579	0.5	(0.4 to 0.5)	111	68	335
**Z – Social problems**	538	0.4	(0.4 to 0.5)	103	29	200
**B – Blood, lymphatics, spleen**	297	0.2	(0.2 to 0.3)	57	31	134
**Unknown**	12 749	10.1	(10 to 10.3)	2 450	551	7 242
**All green encounters**	125 97	100.0		24 207	20 408	37 023
