# Supplementary material for: Reasons for encounter by different levels of urgency in out-of-hours emergency primary health care in Norway: a cross sectional study
Source: BMC Emerg Med. 2017 Jun 24;17:19. doi: 10.1186/s12873-017-0129-2 (PMC5483255; doi:10.1186/s12873-017-0129-2)
Supplement: Supplementary file 3 — Reasons for encounter, ICPC-2 chapters, yellow urgency level (urgent). Counts, proportions. Total, minimum and maximum incidence in individual OOH casualty clinics. (PDF 80 kb) [file 12873_2017_129_MOESM3_ESM.pdf]

Additional table 3: Reasons for encounter, ICPC-2 chapters. Yellow urgency level (urgent). Counts, proportions. Total, minimum and maximum incidence in individual OOH casualty clinics.

		Proportion of yellow RFEs		Incidence (per 100 000 inhabitants per year)		
**ICPC-2 Chapter (RFE)**	**N**	**%**	(95% CI)	**All OOH clinics**	Min	Max
**L – Musculoskeletal**	7 894	17.5	(17.1 to 17.8)	1 517	1 139	2 540
**A – General and unspecified**	6 401	14.2	(13.9 to 14.5)	1 230	1 079	2 468
**D – Digestive**	5 529	12.3	(12.0 to 12.6)	1 062	880	1 980
**R – Respiratory**	5 372	11.9	(11.6 to 12.2)	1 032	730	1 835
**S – Skin**	4 438	9.8	(9.6 to 10.1)	853	636	2 144
**N – Neurological**	2 816	6.2	(6.0 to 6.5)	541	481	772
**P – Psychological**	2 255	5.0	(4.8 to 5.2)	433	398	609
**U – Urology**	2 139	4.7	(4.5 to 4.9)	411	267	679
**K – Circulatory**	1 771	3.9	(3.7 to 4.1)	340	256	546
**F – Eye**	1 340	3.0	(2.8 to 3.1)	257	209	345
**W – Pregnancy, childbirth, family**	401	0.9	(0.8 to 1.0)	77	23	234
**H – Ear**	288	0.6	(0.6 to 0.7)	55	35	104
**X – Female genital system and breast**	286	0.6	(0.6 to 0.7)	55	27	75
**Z – Social problems**	274	0.6	(0.5 to 0.7)	53	0	64
**Y – Male genital system**	271	0.6	(0.5 to 0.7)	52	23	61
**T – Endocrine, metabolic and nutritional**	249	0.6	(0.5 to 0.6)	48	36	84
**B – Blood, lymphatics, spleen**	112	0.2	(0.2 to 0.3)	22	16	35
**Unknown**	3 296	7.3	(7.1 to 7.5)	633	150	1 521
**All yellow encounters**	45 132	100.0		8 673	7602	13 781
