# Supplementary material for: Reasons for encounter by different levels of urgency in out-of-hours emergency primary health care in Norway: a cross sectional study
Source: BMC Emerg Med. 2017 Jun 24;17:19. doi: 10.1186/s12873-017-0129-2 (PMC5483255; doi:10.1186/s12873-017-0129-2)
Supplement: Supplementary file 4 — Reasons for encounter, ICPC-2 chapters, red urgency level (life threatening). Counts, proportions. Total, minimum and maximum incidence in individual OOH casualty clinics. (PDF 79 kb) [file 12873_2017_129_MOESM4_ESM.pdf]

Additional table 4: Reasons for encounter, ICPC-2 chapters. Red urgency level (life threatening). Counts, proportions. Total, minimum and maximum incidence in individual OOH casualty clinics.

		Proportion of red RFEs		Incidence (per 100 000 person-years)		
**ICPC-2 Chapter (RFE)**	**N**	**%**	(95% CI)	**All OOH clinics**	Min	Max
**A – General and unspecified**	1 923	34.4	(33.1 to 35.6)	369.5	136	640
**K – Circulatory**	1 018	18.2	(17.2 to 19.2)	195.6	138	518
**R – Respiratory**	578	10.3	(9.5 to 11.1)	111.1	57	203
**N – Neurological**	547	9.8	(9.0 to 10.6)	105.1	52	305
**D – Digestive**	303	5.4	(4.8 to 6.0)	58.2	22	122
**P – Psychological**	287	5.1	(4.6 to 5.7)	55.2	30	70
**L – Musculoskeletal**	190	3.4	(2.9 to 3.9)	36.5	17	73
**S – Skin**	93	1.7	(1.3 to 2.0)	17.9	5	30
**T – Endocrine, metabolic and nutritional**	50	0.9	(0.6 to 1.1)	9.6	0	18
**W – Pregnancy, childbirth, family**	44	0.8	(0.6 to 1.0)	8.5	2	41
**F – Eye**	27	0.5	(0.3 to 0.7)	5.2	2	30
**X – Female genital system and breast**	16	0.3	(0.1 to 0.4)	3.1	0	10
**Z – Social problems**	16	0.3	(0.1 to 0.4)	3.1	0	12
**Y – Male genital system**	14	0.3	(0.1 to 0.4)	2.7	0	5
**B – Blood, lymphatics, spleen**	11	0.2	(0.1 to 0.3)	2.1	0	10
**U – Urology**	11	0.2	(0.1 to 0.3)	2.1	0	6
**H – Ear**	3	0,1	(0.0 to 0,1)	0,6	0	2
**Unknown**	461	8,2	(7,5 to 9.0)	88,6	15	204
**All red encounters**	5 592	100,0		1 075	570	2 163
